# Very high resolution, altitude-corrected, TMPA-based monthly satellite precipitation product over the CONUS

**DOI:** 10.1038/s41597-020-0411-0

**Published:** 2020-03-03

**Authors:** Hossein Hashemi, Jessica Fayne, Venkat Lakshmi, George J. Huffman

**Affiliations:** 1 Department of Water Resources Engineering & Center for Middle Eastern Studies, Lund University, Lund, Sweden; 2 Department of Geography, University of California-Los Angeles, Los Angeles, California USA; 3 Department of Engineering Systems and Environment, University of Virginia, Charlottesville, Virginia USA; 4 Mesoscale Atmospheric Processes Laboratory, NASA Goddard Space Flight Center, Greenbelt, Maryland USA

**Keywords:** Hydrology, Atmospheric science

## Abstract

The Tropical Rainfall Measuring Mission (TRMM) Multisatellite Precipitation Analysis (TMPA) product provided over 17 years of gridded precipitation datasets. However, the accuracy and spatial resolution of TMPA limits the applicability in hydrometeorological applications. We present a dataset that enhances the accuracy and spatial resolution of the TMPA monthly product (3B43). We resample the TMPA data to a 1 km grid and apply a correction function derived from the Parameter-elevation Regressions on Independent Slopes Model (PRISM) to reduce bias in the data. We confirm a linear relationship between bias and elevation above 1,500 meters where TMPA underestimates measured precipitation, providing a proof-of-concept of how simple linear scaling can be used to augment existing satellite datasets. The result of the correction is the High-Resolution Altitude-Corrected Precipitation product (HRAC-Precip) for the CONUS. Using 9,200 precipitation stations from the Global Historical Climatology Network (GHCN), we compare the accuracy of TMPA 3B43 versus the new HRAC-Precip product. The results show an improvement of the mean absolute error of 12.98% on average.

## Abstract (summary)

**Table:** 

Measurement(s)	hydrological precipitation process
Technology Type(s)	digital curation • computational modeling technique
Factor Type(s)	temporal interval
Sample Characteristic - Environment	weather
Sample Characteristic - Location	contiguous United States of Americ

Machine-accessible metadata file describing the reported data: 10.6084/m9.figshare.11823549

## Background & Summary

The use of the satellite precipitation products in environmental applications has been limited by their accuracy and spatial resolution. Despite the availability of the 0.25° precipitation data from Tropical Rainfall Measuring Mission (TRMM)^1^ available at various temporal resolutions, the TRMM Multisatellite Precipitation Analysis (TMPA)^2^ product is not fully useful for some basin scale and regional hydrometeorological applications. This issue is related to both its accuracy and spatial resolution^3–6^. This limitation is more severe in mountainous regions and transition regions between low and high altitude due to the orographic effect on rainfall rates^7,8^. Studies have shown that TMPA precipitation products can be less accurate over the high elevation and mountainous terrain^9–12^. The inaccuracy of the satellite-based precipitation estimate over the mountainous terrain is due to the high spatial variability of precipitation combined with the low spatial resolution of retrievals from the TRMM multisatellite sensors, as well as systematic biases introduced by sensor technology, precipitation type, applied algorithm, and temporal sampling.

The native TMPA spatial resolution, 0.25° or ~27 km near the equator smooths precipitation peaks^13^ within the grid cell. Further, the passive microwave TRMM multisatellite sensors are not able to observe the orographic enhancement in the liquid phase over the mountainous region leading to underestimation of the actual rainfall, causing a bias in satellite observation^14^. At the watershed scale, data at a high spatial resolution, i.e., <0.1°, are necessary to capture the environmental variability that can be lost at lower resolutions^15,16^. When users choose monthly satellite-based precipitation data as an input in hydrological analyses, lower-resolution data result in a major discrepancy between the measured and simulated runoff^17,18^. Studies suggest that satellite-based products underestimate high rainfall, which is a key parameter for major flooding in a given watershed, introducing a large error in the calibration process of the hydrological model or assessment^9,19,20^. A significant relationship between the high mountainous terrain and precipitation bias from TMPA has been identified in a previous study in which TMPA underestimates the ground-based precipitation measurement over the high elevations—greater than 1,500 meters above mean sea level (amsl)—in the conterminous United States (CONUS)^13^. The same study noted that bias in the satellite data over the high elevation is mainly due to the frequent snow occurrences, particularly in the cold seasons. As the monthly TMPA (3B43) utilizes infrared-based estimates alongside a small number of high altitude gauge measurements to calibrate the precipitation rates, 3B43 tends to underestimate the true precipitation. To offset the underestimated precipitation rates, a previous study developed a correction model for TMPA using the topographically corrected Parameter-elevation Regressions on Independent Slopes Model (PRISM) as a reference^21,22^. The correction model incorporates seasonality and topographically dependent corrections inherent in PRISM, such as coastal proximity, aspect, vertical layer, and topographic position, complemented by an external reference elevation model from USGS GTOPO30^23^. A digital elevation model is required under the assumption that remaining biases are dependent on elevation, and the model was subsequently aggregated to match the 0.25° TMPA-3B43 data to correct the satellite precipitation data. Although the results showed a substantial improvement on the 3B43 product^13^, the coarse resolution of 0.25° is not considered sufficient for applications at local and regional scales.

To improve the TMPA 3B43 product for broader hydrological applications, we present a very high-resolution (~1 km) bias-corrected satellite-based monthly precipitation data set that we name High Resolution Altitude Corrected Precipitation (HRAC-Precip). HRAC-Precip is free and publicly available via NASA’s Goddard Earth Science Data Information Services Center (GES-DISC) https://disc.gsfc.nasa.gov/datasets/HRAC_Precip_V1/summary ^24^. This product demonstrates a proof-of-concept that orographic impacts to satellite precipitation can be corrected using simple linear scaling in relationship with season and elevation. In particular, from an applications perspective, we believe that water resources managers and agricultural land use applications mangers would benefit from the 1-km monthly data, aligning with high resolution land cover datasets. Future work on satellite precipitation correction includes expanding the data to global coverage and producing higher temporal resolution data. The HRAC-Precip monthly product covers the conterminous United States for 1998–2014. Sections 2 and 3 of this of paper explain the data and methods used to produce the HRAC dataset. Validation of the data using 9,200 rain gauge stations is explored in section 4, followed by the conclusion and explanation of data usage in section 5.

## Methods

### Data sources

#### Satellite-based precipitation

TRMM is a collaboration between the National Aeronautics and Space Administration (NASA) and the Japan Aerospace Exploration (JAXA) Agency to estimate precipitation and lightning over the tropical and subtropical regions of the globe (latitudes 40° North to 40° South). TRMM was launched in November 1997 and ended its mission in April 2015. The TRMM platform carried five different sensors of which three sensors, precipitation radar, microwave imager, and visible infrared scanner, were used to estimate precipitation. TMPA^1,2^ is a merged satellite-gauge product that provides three-hourly precipitation dataset between latitude 50° North and 50° South and utilized the retrievals from satellite systems in the TRMM precipitation satellite constellation. The TMPA 3B43 data is a monthly research-oriented product that is an aggregation of the 3-hourly product with gauge data where applicable. The gauge data used in TMPA data production is 1° gridded precipitation from the Global Precipitation Climatology Centre (GPCC)^25,26^. In this study, we used the TMPA 3B43 monthly precipitation product for the period January 1998 to December 2014.

#### Ground-based precipitation

**Fig. 1:**
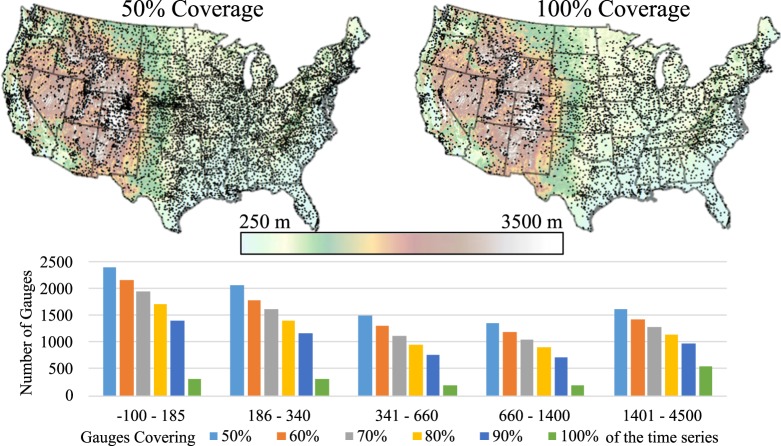
Study area and rain gauge network showing different temporal and spatial coverage. The study area contains approximately 9,200-gauge stations spanning the period 1998–2014. The maps show the stations that cover 50% and 100% of the time series. The bar graphs illustrate the numbers of gauges with different coverages of the time period for five different ranges of elevation (elevations given in m).

Ground-based station data are required for validation of the proposed methodology and were collected from the Global Historical Climatology Network (GHCN) Global Summary of the Month (GSOM)^27^, a database containing ~51,000 historical monthly precipitation stations in the United States with additional stations outside of the United States. The large database was filtered with the requirement that the data in the time period January 1998–December 2014 should contain at least 50% of the months within the studied time period, 102 out of 204. The data were further divided into categories where stations contained at least 60, 70, 80, 90, and 100% monthly observations within the time period. Figure 1 shows the stations with at least 50% temporal coverage, totaling 9,243, while the 100% coverage category contains 1,574 stations.

#### Digital elevation model (DEM)

As the goal of the study was to provide an elevation corrected high-resolution monthly precipitation product, the spatial resolution of the digital elevation model was chosen to be 1 km, providing the structure for the correction to produce a 1 km precipitation dataset. The Global 30 Arc-Second Elevation (GTOPO30)^23^ DEM dataset was selected for its high spatial resolution, affording the production of the high-resolution precipitation product. As the data are provided by the United States Geological Survey in a tiled format, several large tiles were initially merged into a single mosaic providing a complete DEM of the CONUS and neighboring regions, then clipped into a region representing only the CONUS. The data temporal availability and spatial resolution are summarized in Table 1.

**Table 1:** Summary of the precipitation and elevation datasets used in this study.

Source	Product	Grid size	Time span
Surface gauge-based dataset	GHCN	Point measurement	1998–2014
Satellite-based dataset	TMPA 3B43	0.25°	1998–2014
DEM dataset	GTOPO30	0.01°	—
New satellite-based dataset	HRAC-Precip^24^	0.01°	1998–2014

The bar graph in Fig. 1 tallies station coverage at varying elevation bands summarizing the spatial distribution of the stations. The DEM was divided into five equal-area regions, with each elevation band covering ~20% of the contiguous region, and relating the station locations by area coverage and elevation simultaneously.

### Application of methodology

The goal of this study was to produce a high-resolution monthly satellite data based on the correction model proposed by Hashemi *et al*.^13^. The previous study developed a correction model that reduced the mean absolute error of the satellite bias at the elevations above 1,500 m amsl by 5.4% across all seasons. The correction is applied to elevations above 1,500 m amsl because biases are negligible below this elevation; lower elevations are not susceptible to orographic lifting and therefore underestimation by the microwave sensors. Thus, the primary assumption of the model is that the bias has a strong dependence on elevation and topography. The correction model (Eq. 1) is a linear function and assumes the dependence of relative bias in the satellite data, at the pixel level, on elevations above 1,500 m amsl (Eq. 1).

1 $${S}_{ic}={S}_{i}(\alpha \,{E}_{i}+\beta +1)$$

where subscript *i* is the specific pixel, *S*_*ic*_ is the corrected satellite data, *S*_*i*_ is the original satellite estimate, *E*_*i*_ is the elevation, *α* and *β* are two constants and 1 is a normalizing factor derived from the relative bias equation, discussed in Hashemi *et al*.^13^. As elevation is considered to be the primary bias producing factor, model coefficients are computed by randomly selecting a subset of the precipitation data during the calibration procedure along with the elevation of the selected pixel. The calibration of the model and coefficient estimation are carried out at the pixel level using the Monte Carlo Cross Validation technique for each month. Computed coefficients are then validated against the topographically corrected Parameter-elevation Regressions on Independent Slopes Model (PRISM)^21,28^ estimates, ensuring that the corrected data correctly incorporates topographically dependent adjustments inherent in PRISM, such as wind direction, aspect, topographic position, and others. For more detail on the algorithm used to produce the coefficients and original validation, refer to Hashemi *et al*.^13^.

We applied the correction model (Eq. 1) to the resampled high-resolution satellite data at the pixel level for each month corresponding to the appropriate monthly coefficients (Table 2). While the coefficients are uniform for each month across all pixels, variations in the elevation from the DEM along with the coefficients produce new precipitation values.

**Table 2:** Monthly correction model coefficients. As computed by Hashemi *et al*.^13^, model coefficients were derived by using a Monte Carlo Cross Validation approach with input from four randomly selected years for the elevations above 1,500 m amsl.

Month	Jan	Feb	Mar	Apr	May	June	July	Aug	Sep	Oct	Nov	Dec
*a* (×10^−3^)	0.7316	0.8422	0.6415	0.4654	0.2078	0.1372	0.2642	0.2458	0.2344	0.3299	0.6928	0.7785
*β*	−1.3202	−1.4827	−1.0475	−0.7371	−0.2777	−0.2639	−0.4941	−0.4266	−0.3592	−0.4655	−1.1823	−1.3022

By applying Eq. 1 to 3B43, Hashemi *et al*.^13^ were able to significantly reduce the bias in the high mountainous terrain of the CONUS relative to the ground-based estimate.

**Fig. 2:**
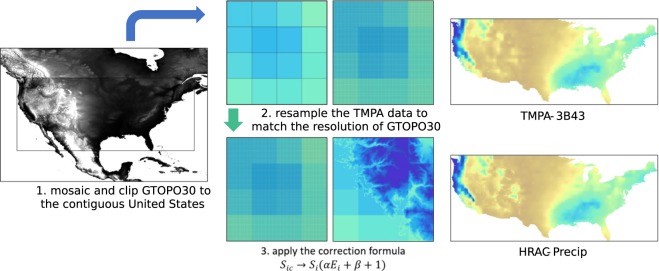
Flowchart summarizing the methodology implemented in this study. 1.) 30 arc-second digital elevation model, 2.) TMPA-3B43 resampled into the DEM data (~1 km), and 3.) the correction model was applied to the downscaled TMPA-3B43 to produce high-resolution altitude-corrected TMPA 3B43 (High Resolution Altitude-Corrected Precipitation HRAC-Precip).

While the previous study corrected the TMPA product using the native 0.25° spatial resolution with an aggregated DEM, this study disaggregates the TMPA data, allowing the DEM to retain its original spatial resolution and providing increased level of detail in the corrected precipitation. As the precipitation bias is sensitive to elevation, aggregated elevations have the potential to skew orographic precipitation estimates, since elevation may be higher or lower contributing to local changes in cloud uplift. Here, we chose a DEM with a horizontal grid spacing of 30 arc-second (~1 km, or 0.0083°). The methodology is illustrated in Fig. 2 and explained in the following sections.

#### Interpolation/resampling and spatial resolution

To produce the High Resolution Altitude-Corrected Precipitation (HRAC-Precip), the original 3B43 product was resampled into the GTOPO30 DEM grid size (~1 km). This was done using nearest neighbor resampling to preserve all of the original values from 3B43. Each 0.25° pixel was subdivided into approximately 900 smaller 0.0083° (30 arc-second) pixels, each containing the original 0.25°-pixel value, representing the original spatial average for the region. This provides a precipitation dataset on the DEM spatial grid.

#### Bias calculation and correction

To measure the differences between the satellite and gauge before and after the correction, we calculated the relative bias (*δb*_*i*_) between the high-resolution satellite data and the rain gauge measurement, pixel to point, for the entire country using Eq. 2:

2 $$\delta bi=2\frac{{S}_{i}-{R}_{i}}{e+({S}_{i}+{R}_{i})}$$

where *R*_*i*_ is the rain gauge measurement and the denominator, *e*, contains a small value (15 mm/month, or 0.5 mm/day) to normalize low precipitation amounts and reduce large relative bias from low-precipitation events.

**Fig. 3:**
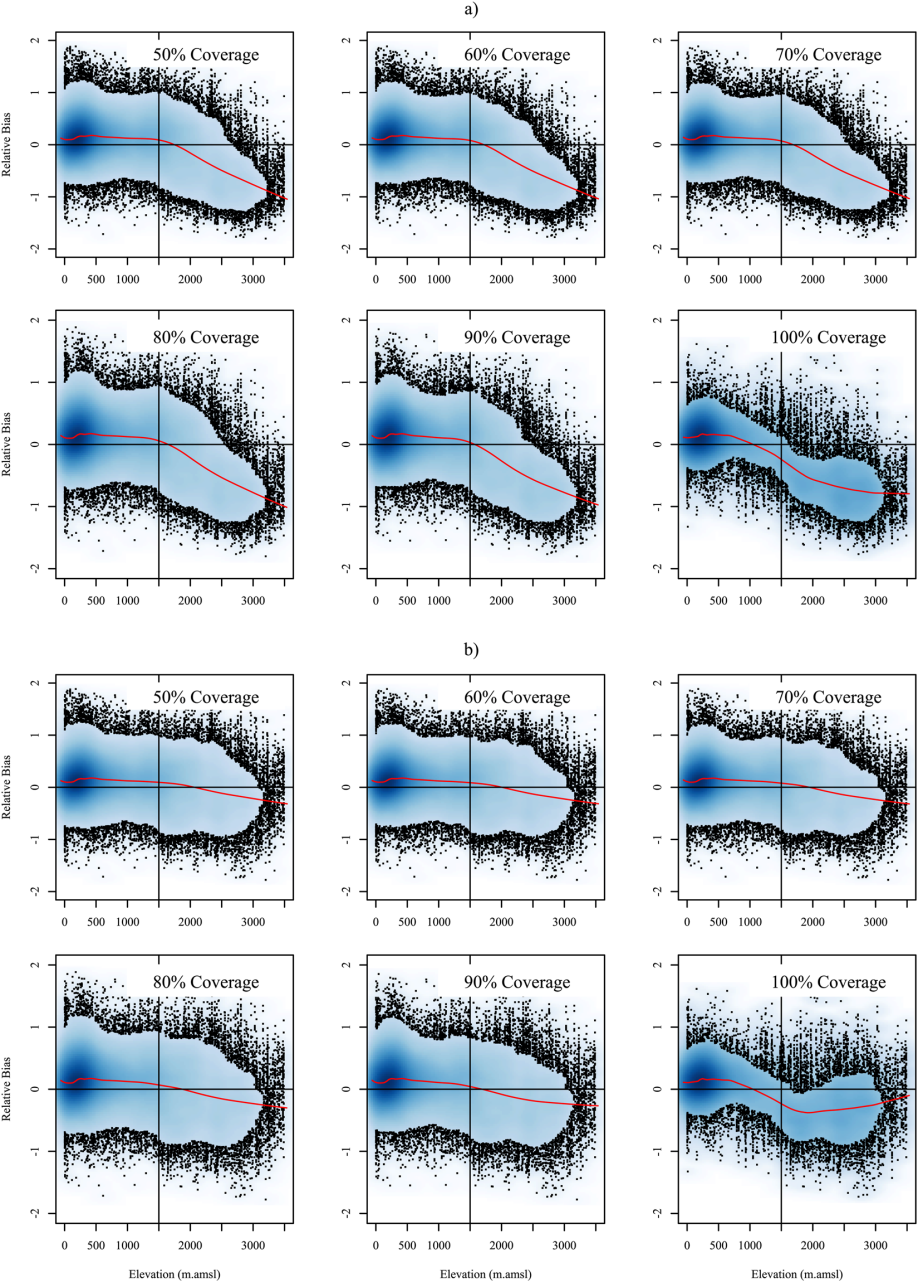
Scatter plots depicting the relationship between measurement bias and elevation. The top two rows (**a**) show the average relative bias of the Decembers in 1998–2014 between the satellite product and the rain gauges matching 50-100% of the satellite period. The bottom two rows (**b**) show the relative bias between the elevation-corrected satellite product and rain gauges matching 50-100% of the satellite period (1998–2014). A horizontal line is drawn at zero bias, with a vertical line drawn at 1,500 m amsl. All black dots shown in the graph represent stations, and the scatterplot is smoothed where the plotted points are densely clustered. A local polynomial regression line is shown in red, highlighting the relative bias trend with elevation.

To validate the corrected high-resolution satellite data, we compared the new product with rain gauge measurements across the CONUS during 1998–2014 using the GHCN precipitation station dataset^27^. We assumed that each of the ~9,200 gauges represent an area equivalent to the 1 km pixel size. Based on the elevation classification (Fig. 1), only about 20% of the area of the CONUS is occupied by the high elevations (>1,500 m) for which we applied the correction model. As in the previous study, we did not apply any correction to the lower elevation bins, as the biases observed in TMPA-3B43 below 1,500 m amsl were very small. We compared each HRAC-Precip pixel with a corresponding gauge across the CONUS, with gauges overlapping of 50, 60, 70, 80, 90, and 100% of the time with the HRAC product. The comparison of the relative bias with the point elevations are shown in Fig. 3, demonstrating the topographic elevation dependent bias.

## Data Records

Data used in the production of the new HRAC-Precip product are described in the Methods: Data Sources section of this manuscript. The final HRAC-Precip product^24^ is available via GES DISC (https://disc.gsfc.nasa.gov/datasets/HRAC_Precip_1). R programming language and Matlab scripts^29^ are used to produce (Eq. 1) and validate (Eq. 2) this data as well as the Monte Carlo coefficient analysis and are publicly available through GitHub: https://github.com/JVFayne/HRAC-Precip_v1. Due to the simplicity of the correction formula, the scripts can be easily translated to other programming languages.

## Technical Validation

We calculated the relative bias (Eq. 2) between the HRAC-Precip and the rain gauge measurements across CONUS. Figure 3a depicts the average relative bias for all Decembers during 1998–2014 between the uncorrected 3B43 and rain gauge measurements against elevation in CONUS. To include as many as gauges as possible in the analysis, we calculated the relative bias including the gauges in which they had 50 to 100% temporal overlap (Fig. 1) with the satellite data period. The number of gauges at the highest elevation bin decreases from over 1,500 to about 500 gauges for 50 to 100% temporal coverage, respectively (Fig. 1). As can be seen in Fig. 3a, the high-resolution satellite product underestimated the ground-based measurements in the high elevations of the CONUS. Local polynomial regression lines are used to summarize the relationship between the relative bias and the elevation. The local regression method “pulls” the regression line to where points are the densest, allowing for a more detailed view of the relationship between the relative bias and the elevation, particularly if the relationship is not perfectly linear^30^. The local regression lines for the relative bias in the temporal coverage (50–100%) shows a very small bias for elevations below 1,500 m amsl, while the error increases sharply at higher elevations, mainly above 2,500 m amsl, in all temporal overlaps. It is important to note that since 3B43 applies a wind-induced under-catch correction to the GPCC surface gauge analysis, the average precipitation estimate by the satellite-gauge combination is expected to be higher than the rain gauge measurements for elevations below 1,500 m amsl.

In all cases, the calculated bias showed a linear dependence on the elevation where the negative bias increased with the elevation above 1,500 m amsl. However, the negative bias for the 100% match is noticeable at elevations lower than 1,500 m amsl, which is due to fewer gauges in the elevation bin 1,000–2,000 m, skewing the local regression line below zero bias at the elevation around 1,000 m.

The result is in agreement with the previous study conducted by Hashemi *et al*.^13^ with regards to the linear dependence of the bias on elevation using the native 0.25° TRMM 3B43 pixel size. To correct for this bias, we applied the same correction model and calculated monthly coefficients as suggested by Hashemi *et al*.^13^ to the resampled 1 km 3B43 data. We only applied the correction model to the pixels covering the elevation above 1,500 m amsl, hence the low elevation precipitation pixels remained unchanged due to the negligible bias relative to the gauge measurements. Figure 3b depicts the scatter plots of the average relative bias between the HRAC-Precip and rain gauge measurements against the elevation for the same month as Fig. 3a. Figure 3b demonstrates the improvement of the relative bias from almost 100% to less than 20% across all temporal coverage between satellite and rain gauges. The results confirm that the suggested correction model is quite robust and the bias in the satellite is primarily dependent on elevation.

Using the stations covering 100% of the study period, the Root Mean Square Error (RMSE) and the Mean Absolute Error (MAE) was summarized by season and elevation above 1,500 m before and after correction (Table 3). These results demonstrate that the corrective method notably improved error estimates by an average RMSE of 11.44% and an average MAE of 12.98% across all seasons, indicating a substantial improvement over the original method in Hashemi *et al*.^13^, where MAE improvement averaged 5.4% across all months. The improvement between studies may be due to the increased precision of the point-based gauge stations over the ~25 km gridded gauges and producing the higher spatial resolution HRAC-Precip product.

**Table 3:** Root Mean Square Error and Mean Absolute Error statistics. Statistics are calculated only using the stations that cover the entire study period (1998–2014).

Season	TMPA (3B43)		HRAC-Precip	
	RMSE	MAE	RMSE	MAE
Spring	37.2	28.49	33.8	25.25
Summer	23.07	16.52	21.67	15.63
Fall	56.19	47.51	48.4	40.13
Winter	52.49	42.1	43.72	33.84

**Fig. 4:**
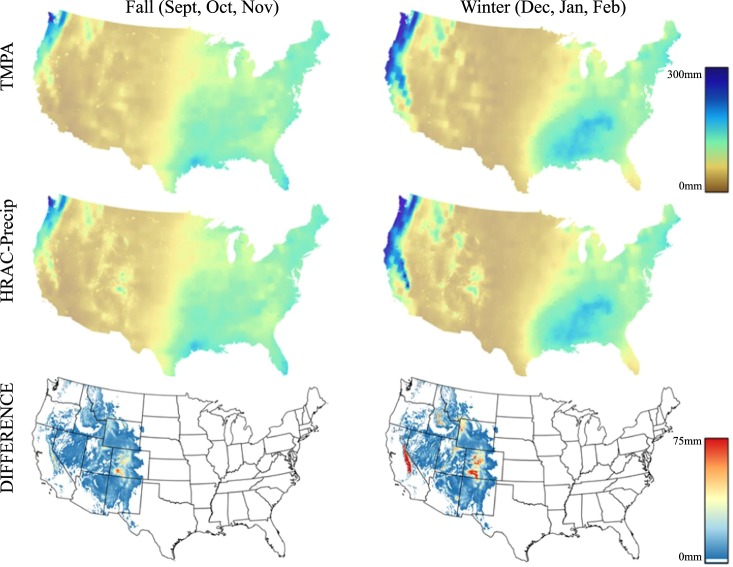
Average seasonal precipitation from TMPA and HRAC-Precip. Precipitation estimates from TMPA (first row), the HRAC-Precip (second row), and their difference as [HRAC-Precip – TMPA] (third row).

Figure 4 depicts the average seasonal precipitation estimated by 3B43 for fall and winter seasons during 1998–2014 in comparison with the HRAC-Precip product. It is evident that HRAC captured more precipitation detail in the high mountainous terrain of the western CONUS relative to the original 3B43. As the bias correction increases with elevation, higher elevations will have the highest amount of correction. The amount of the correction applied to the satellite data varied between a few millimeters to about 75 mm, as can be seen in the Sierra Nevada Mountains in California and the Rocky Mountain in Colorado, due to the linear elevation-dependent formula.

**Fig. 5:**
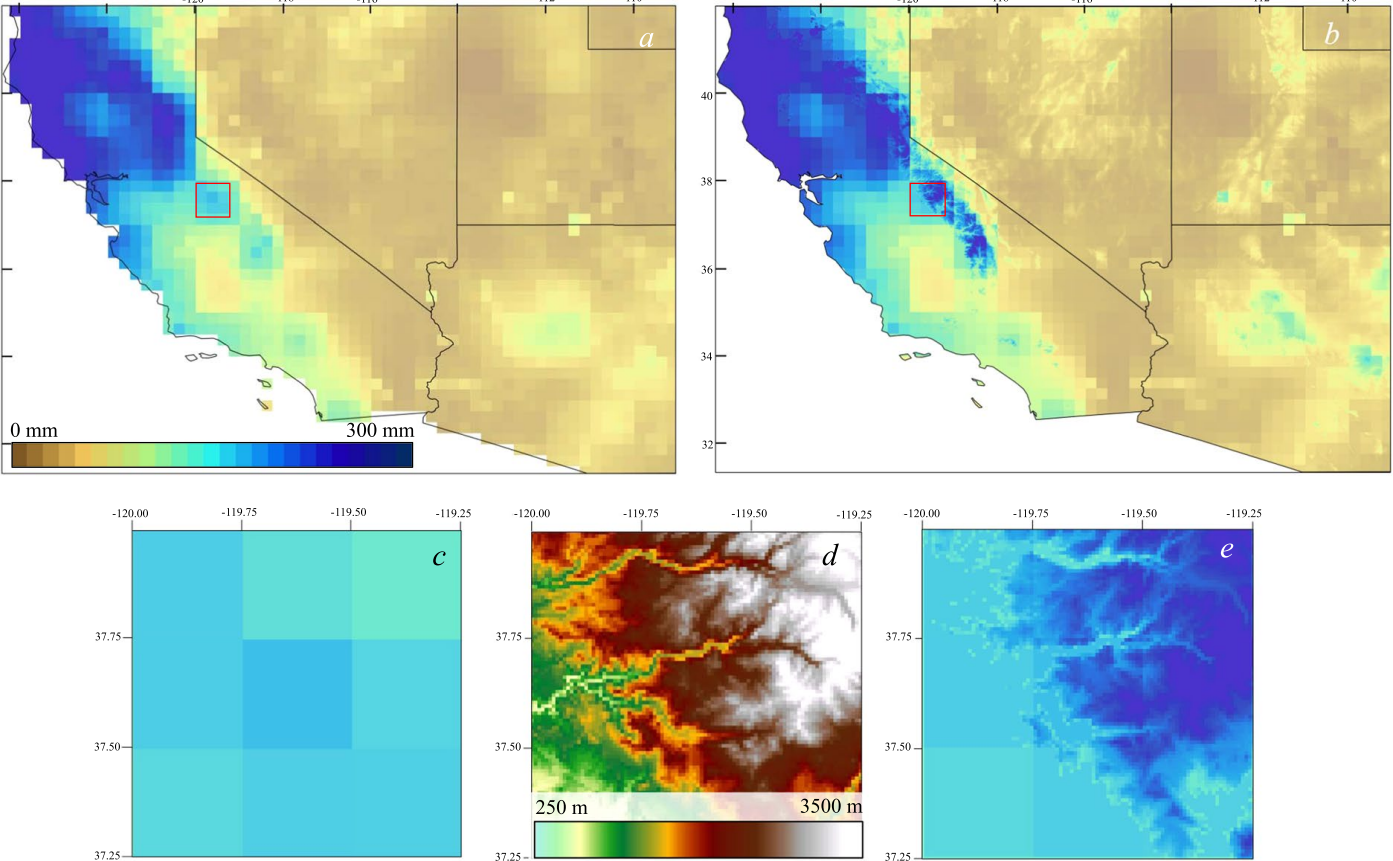
A mapped comparison of TMPA and HRAC-Precip focusing on the Sierra Nevada Mountains. The TMPA precipitation (**a**) for the winter composite (as in Fig. 4) in comparison with the new high resolution HRAC-Precip (**b**). The small region of the Sierra Nevada Mountains enclosed by the red box in (**a**) and (**b**) is enlarged to show nine pixels of the original 3B43 (**c**), the 30 arc-second DEM (**d**), and the HRAC-Precip (**e**).

To illustrate the differences between the original TMPA 3B43 and the HRAC-Precip, Fig. 5 shows the precipitation amount for the winter composite over a small region in the Sierra Nevada Mountains in California. We picked a mountainous area within a 5,625 km^2^ region, representing nine TMPA 3B43 pixels (0.25°; Fig. 5a,c) and compared with that of the HRAC-Precip (Fig. 5b,e). As Fig. 5 shows, the new product provided a higher level of detail in the mountainous region of the west relative to the original 3B43. For the selected region, TMPA 3B43 produced nine pixels (Fig. 5c) with different precipitation values while the new product delivered ~8,100 precipitation values (Fig. 5e), providing more information about the precipitation over the same mountainous area. Where the altitude is lower than 1,500 m amsl (Fig. 5d), the original 3B43 values are present.

## Usage Notes

High-resolution gridded precipitation product provide a valuable basis for hydrometeorology applications in the mountainous regions. However, their accuracies need to be validated^31^. Precipitation products from the TMPA have made a significant contribution to the field of hydrometeorological studies in the past two decades. Nevertheless, the use of TMPA in the local and regional studies has been challenging due to the spatial resolution and accuracy in the mountainous regions. Toward this end, motivated by previous work by Hashemi *et al*.^13^, we built a simple methodology to simultaneously improve the accuracy and spatial resolution of monthly TMPA version 3B43, over the CONUS for the years 1998–2014. This new HRAC-Precip data product is available via GES DISC (https://disc.gsfc.nasa.gov/datasets/HRAC_Precip_1) in the NetCDF-4 format.

Using the Hashemi *et al*.^13^ result that the satellite underestimation in the high mountainous region of western CONUS is related to the elevation, the correction model assumes a linear relationship between bias and elevation, and substantially reduces bias in the satellite data in the elevation above 1,500 m amsl. Since elevation datasets are available at various resolutions, we resampled the TMPA-3B43 into the 1 km grid matching the reference elevation dataset from GTOPO30 and applied the correction model to the new high-resolution interpolated 3B43, thereby producing HRAC-Precip. We compared the original satellite data as well as the new corrected product to the monthly precipitation measured at ~9,200 rain gauges across the country. The results showed a significant improvement in both accuracy and spatial resolution of the satellite data.

As high elevation areas are targeted for positive correction, there are some instances of positive biases becoming more positive. However, the comparison of the mean absolute error (Table 3 Winter MAE: 42.1 → 33.84) demonstrates that the positive biases are not increasing at the same rate that the negative biases are being reduced, and that the correction method notably decreased the MAE, rather than merely shifting values into a positive bias. We believe that this methodology can be used across the high mountainous regions of the CONUS, where the gauge measurements are scarce, to improve the satellite precipitation data for hydrometeorology applications by providing very high-resolution gridded precipitation data at no cost and with high accuracy.

The entire dataset has been resampled to a higher spatial resolution to facilitate the correction in higher elevations with complex topography. The areas that have been modified are limited to the western United States, as seen in Fig. 4, meaning the data in lower elevations that are dominant in the mid-west and the east will not differ from the original TMPA dataset. Thus, it is likely that studies using this dataset will focus in the western continental United States. Because the dataset is made at a higher spatial resolution, users are encouraged to apply it to regional analysis as they would using the original TMPA. Furthermore, finer basin-scale hydrological assessments^32^ are made possible by the higher spatial resolution. In particular, we expect that this data will be used for water resources analysis and will be integrated with other high-resolution datasets such as remotely senses land cover and soil moisture data. In addition to HRAC, other gridded precipitation products over the United States are also available for the same period, such as PRISM used in this study to produce HRAC-Precip, GPCC used to produce TMPA, as well as CMORPH, PERSIANN, and many other gridded precipitation produced reanalysis methods. Each of these datasets are unique in how they were produced, with inherent biases. Scientific users are strongly encouraged to assess which of the many data products available are most appropriate for their study region and use case.

## Data Availability

R programming language and Matlab scripts^29^ used to produce (Eq. 1) and validate (Eq. 2) this data as well as the Monte Carlo coefficient analysis are publicly available with a public access license through GitHub: https://github.com/JVFayne/HRAC-Precip_v1. Due to the simplicity of the correction formula, the scripts can be easily translated to other programming languages; the free to use open source packages ‘raster’, ‘rgdal’, and ‘rgeos’ are required to use the R scripts, although the code functions of these packages that are used in the scripts (such as reading and writing geospatial files) do not change over the course of version updates, and many other programming languages such as Matlab and Python use similar packages to read and write raster files. Additional software packages are not required to produce these data.
